# Book Reviews

**DOI:** 10.1038/sj.bjc.6690672

**Published:** 1999-09

**Authors:** David Gilligan

**Affiliations:** Papworth Hospital, NHS Trust Papworth Everard,Cambridge, CB3 8RE


British Journal of Cancer (1999) 81(1), 187–188
© 1999 Cancer Research Campaign
Article no. bjoc.1999.0671

### Book Reviews

### Cancer Chemotherapy Treatment Protocols

Khayat, Waxman and Antoine, Publication details:
Price £24.95, ISBN 0–86542–693–7. Blackwell Science;
UK, 1998.

The authors of this text should be applauded for aiming to provide a comprehensive chemotherapy protocol book and easy reference text for the practicing oncologist. Do they achieve their goal? They have certainly provided a broad compilation of the majority of commonly used chemotherapy protocols for malignant disease, which will serve as a good reference text in oncology departments.

The book has an interesting and unusual style and format. There is a short introductory section entitled ‘General Principles’, which covers supportive therapy, and toxicity and administration guidelines. I found this section suffered from being too brief. The authors should have been able to cover toxicity and administration for all the commonly used cytotoxic drugs in some detail. In the supportive therapy section, there are some idiosyncrasies of treatment with which I would not necessarily agree. Unfortunately, because the book is not referenced in the conventional way, it is not possible to access the source easily and examine the evidence for the guidelines. For example, in the treatment of extravasation of vesicant cytotoxic drugs, the authors suggest using 99% dimethyl sulphoxide four times daily for 2 weeks and reference a randomized study in rats. These recommendations would certainly not be current standard practice in oncology centres, but if it is true, we need to see the evidence for it so that improvements in practice can be introduced. For the commonly used drugs some important information is emphasized, which will guide good clinical practice, but in my view, this section should have been more comprehensive.

The main section of the book itemises the majority of protocols used over the past 25 years. In the larger chapters (e.g. on breast cancer) it is difficult to discover how the protocols have been ordered, although the sequence seems to be more or less chronological. It would be helpful to know whether there is anything more significant about the order in which the protocols are presented, because it would enable the reader to give some sort of ‘weighting’ to each. The comments sections are on the whole very useful, giving information on response rates and some important comparisons with other protocols, together with a reference for each protocol.

There are some inaccuracies and omissions. For example, the AC regimen listed for breast cancer is not the one that most practicing oncologists would recognize or frequently use. The single agent activities listed at the beginning of each chapter are useful but, unfortunately, some contain inaccuracies, e.g. to ascribe cisplatinum a single agent response rate of only 27% in ovarian cancer is incorrect. In the section on osteosarcomas in protocols using high-dose methotrexate, the folinic acid rescue regimens are unclear and, despite the fact this is covered in the introductory section, this is of such importance that I think it should be emphasized within each protocol using high-dose methotrexate. In the section on Ewing’s sarcoma, the now commonly used IVAD/IVA and EVAIA protocols are not included – which they should be for this rare tumour. There are some inaccuracies in the section on non-small-cell lung cancer, soft tissue sarcomas and osteosarcoma which could be a little more serious if attempts were made to implement them by practicing oncologists. Discrepancies between this book and the clinical papers it cites could lead patients to be over- or under-treated.

As a comprehensive reference book and short guide to the majority of chemotherapy protocols, this book works well enough and will find a place as a reference book in oncology pharmacies and oncology libraries, and on the shelves of practicing oncologists. However, despite the disclaimer by the authors and publishers at the start, the inaccuracies that it contains should be addressed.

*Helena Earl*
*Oncology Centre*
*University of Cambridge*
*Box 193 Addenbrooke's Hospital*
*Hills Road*
*Cambridge CB2 2QQ*

### Lung Cancer, Second Edition

Edited by JA Roth, JD Cox and WK Hong, Publication
Details: Price £75.00, ISBN 0–86542–573–6. Blackwell
Science: UK, 1998.

This book is the second edition of a book which aims to cover all aspects of lung cancer. It has been updated over the last 5 years and edited by authors from the MD Anderson Cancer Centre, Texas, USA.

This book would aim to pitch itself as an intermediate work of reference, i.e. it should be a source of information more detailed than a general oncology textbook but fall short of the most recent advances in lung cancer which by their very nature would have to be published in peer reviewed journals. There are 20 chapters that cover most areas of lung cancer. A large number of chapters focus on developing strategies in lung cancer such as molecular and fluorescent detection, chemoprevention, lung cancer genetics and growth factor and biologic approaches to therapy. The more standard treatment modalities for lung cancer, such as surgery, radiotherapy and chemotherapy, are covered but there are some notable omissions which will be discussed later.

The early chapters include the biology of pre-neoplastic regions, and give a very detailed and good historical background to the subject. In particular, there is a good summary of the chromosomal abnormalities present and some discussion on how multistage carcinogenesis may apply in this disease. The chapter on familial predisposition to lung cancer again gives a background to the subject, but illustrates the point that there are as yet no known susceptibility genes.

The next three chapters discuss recent advances in surgery. There is an excellent overview of preoperative evaluation for patients requiring surgery. Although the described preoperative work-up of a patient requiring lung cancer surgery is more elaborate than one would expect for a UK practice, it is becoming apparent that patients will require a more detailed estimation of their lung function prior to surgery and this is well described. It was pleasing to read that the authors are against segmental resection, as most practitioners in this field would feel that a lobectomy would be the minimum required operation. There is an increasing role for keyhole thorascopic surgery in the diagnosis and treatment of lung cancer, and the chapter by Lo Cicero III again gives a balanced view of the do's and don'ts of video-assisted thoracic surgery (VATS). It discusses the very difficult problem regarding sampling of the subcarinal node station. However, Lo Cicero III does fall short of discussing the very useful application of VATS in the treatment of effusions and a diagnosis of mesothelioma. There is a small, but definite, place for surgery for small-cell lung cancer and this is nicely described. The role of surgery should probably be limited to those patients with true stage I disease who have been fully assessed prior to surgery.

The role of radiotherapy is discussed in three chapters. The first deals with endobronchial brachytherapy and highlights the problem that we do not really know enough about this modality to be sure of its place within the therapeutic armamentarium. It would probably prove useful in recurrent disease previously treated with external beam radiotherapy, and possibly as a boost for patients treatable with radical radiotherapy. Some of the illustrations in this section leave much to be desired and are of poor quality. The next chapter deals with conformal and three-dimensional radiotherapy. It suffers from the fact that there are no randomized trial data. Probably one of the most major omissions in the book is that there is no mention of altered fractionation and, in particular, Continuous Hyperfractionated Accelerated Radiotherapy (CHART). This treatment has been shown in a randomized trial to confer a 9% absolute survival benefit and is one of the major advances in the treatment of NSCLC in recent years. Indeed, the next chapter on concurrent chemotherapy and radiation has been shown to confer only very marginal advances over conventional radiotherapy but merits its own chapter.

The chapter on pre- and post-operative adjunctive therapy in resectable NSCLC is perhaps surprisingly short given the intense amount of controversy in this area. The oft quoted studies of Rosell and Roth are mentioned only briefly and not discussed in any great detail. In particular, it is noticeable that the reference for Roth relates to an abstract rather than the definitive paper which has been in general circulation for many years. The results of the MRC meta-analysis of post-operative radiotherapy (PORT) were published too late for inclusion in this book but would make an interesting addition. It also proposes a new staging for stage III disease but it is interesting to note that it does not take into account the revised classification by Mountain in 1997 where T3 N0 tumours are now stage IIB. The authors of this chapter would wish them to be described as stage $III_1$.

The many new agents available for treating NSCLC are described in great detail and as most of these studies are phase II they concentrate purely on response rates. It is also of note that most of the phase III studies do not show any improvement in median survival for stage IIIB and IV disease. One omission, which will become less tolerated in the oncological community as a whole, is the requirement to include quality of life data in evaluating these new agents.

The emerging therapies of chemoprevention and photodynamic therapy are discussed, but their clinical relevance remains uncertain. Other major omissions in this book include no mention of the role of PET scanning in N2 and metastatic disease. The management of small-cell lung cancer, although taking a back seat compared to new studies in NSCLC, does deserve some mention. In particular, discussion of new agents both chemotherapeutic and anti-angiogenic. The role of prophylactic cranial irradiation (PCI) should be discussed in the light of new trials and a meta-analysis.

All in all this book would be a useful addition to an oncology department library and is essential reading for the sub-specialist. I would therefore recommend its purchase to those categories, but I feel it does not warrant wider purchase.

*David Gilligan*
*Papworth Hospital NHS Trust*
*Papworth Everard*
*Cambridge CB3 8RE*

*© 1999 Cancer Research Campaign*

